# Sequencing and Annotation of Duggie and Hocus, Two Subcluster B1 Mycobacteriophages

**DOI:** 10.1128/MRA.00432-20

**Published:** 2020-07-09

**Authors:** Erin L. Doyle, Alexis N. Burke, Samuel J. Coy, Haley R. Miller, Lilly M. Shatford-Adams, Reagan M. Petersen, Kade B. Wehrs, Dane M. Bowder

**Affiliations:** a Department of Biology, Doane University, Crete, Nebraska, USA; Loyola University Chicago

## Abstract

Two mycobacteriophage genomes were newly sequenced and annotated. Duggie and Hocus were discovered, enriched, and isolated from soil using Mycobacterium smegmatis mc^2^155. The bacteriophages are lytic Siphoviridae and belong to the B1 subcluster. The Hocus and Duggie genomes are highly similar to one another in both nucleotide sequence and gene content.

## ANNOUNCEMENT

Bacteriophage genomes were among the first complete genomes sequenced due to their relatively small size and simplicity (1). Studying bacteriophage genomes helps to identify bacteriophages with therapeutic potential, especially in the treatment of antibiotic-resistant bacterial infections (2, 3). In this study, we annotated the genomes of the mycobacteriophages Duggie and Hocus. Duggie and Hocus were found at Doane University in Crete, Nebraska. Soil samples were collected from campus, phages were isolated by enrichment culture and purified by repeated serial dilution, and samples were amplified by generating a lysate using Mycobacterium smegmatis mc^2^155. The host bacteria were grown in 7H9 broth and incubated at 37°C with agitation. Transmission electron microscopy (TEM) imaging revealed that the phages are siphoviruses. Sequencing confirmed that the phages are lytic and belong in the B1 subcluster.

Bacteriophage genomic DNA was isolated using the Wizard DNA extraction kit (Promega) and sequenced at The Pittsburgh Bacteriophage Institute. Sequencing libraries were prepared from genomic DNA using a New England BioLabs (NEB) Ultra II kit with dual-indexed barcoding. Libraries were pooled and run on an Illumina MiSeq instrument, yielding at least 100,000 single-end 150-base pair reads and a 1,057-fold coverage (504,224 raw reads) for Hocus and 1,353-fold coverage (657,250 raw reads) for Duggie. No further quality control or adapter trimming was performed on the reads provided by the sequencer. These raw reads were assembled using Newbler v.2.9 (default settings). Each genome yielded a single contig, which was checked for completeness, accuracy, and phage genomic termini using Consed v.29 as previously described (4).

Autoannotation in DNA Master v.5.23.3 (http://cobamide2.bio.pitt.edu) running GeneMark v.2.8 (5) and Glimmer v.3.02 (6) predicted gene locations. Gene start sites were identified using Starterator (https://seaphages.org/software/), ribosomal binding site (RBS) scores within DNA Master, and BLASTp comparisons to similar bacteriophage genes. Gene functions were assigned using Phamerator (7), HHpred (8), PECAAN (9), and NCBI BLAST (10). ARAGORN v.1.1 (11) was used to confirm the absence of tRNA genes, which are not commonly found in subcluster B1 phages. Characteristics of the two genomes are shown in Table 1. All tools were run using default parameters, unless otherwise stated.

**TABLE 1:** Characteristics of two bacteriophage genomes

Phage name	GenBank accession no.	SRA accession no.	GC content (%)	Sequence length (bp)	No. of genes	No. of genes with assigned functions	Closest relative	Identity with closest relative (%)
Duggie	MN585965	SRX7344740	66.4	68,885	102	31	JakeO	99.59
Hocus	MN369738	SRX7344741	66.4	68,083	100	27	Mutante	98.47

The Duggie and Hocus genomes display nucleotide similarity to each other and to other subcluster B1 phages, as determined by global nucleotide BLAST. Specifically, the genomes of Duggie and Hocus are 98.95% identical to one another. The genome of another B1 phage, Kloppinator, is 95% identical to both the Hocus and Duggie genomes. Notably, Duggie and Hocus differ from Kloppinator in a region between bp 55976 and 57353, where the Kloppinator genome contains a different gene, Kloppinator_71, that is not present in Duggie or Hocus. The Hocus genome includes two genes not found in Duggie or Kloppinator; Hocus_67 is an orpham with no known function, and Hocus_90 belongs to a pham of only six members, all of which are cluster B phages (12).

### Data availability.

Genome sequences and raw sequence data are available in GenBank and the Sequence Read Archive. Accession numbers are shown in Table 1.

## References

[1] Hatfull GF 2008 Bacteriophage genomics. Curr Opin Microbiol 11:447–453. doi:10.1016/j.mib.2008.09.004. 18824125 PMC2706577

[2] Dedrick RM, Guerrero-Bustamante CA, Garlena RA, Russell DA, Ford K, Harris K, Gilmour KC, Soothill J, Jacobs-Sera D, Schooley RT, Hatfull GF, Spencer H 2019 Engineered bacteriophages for treatment of a patient with a disseminated drug-resistant Mycobacterium abscessus. Nat Med 25:730–733. doi:10.1038/s41591-019-0437-z. 31068712 PMC6557439

[3] Hatfull GF 2018 Mycobacteriophages. Microbiol Spectr 6. doi:10.1128/microbiolspec.GPP3-0026-2018. PMC6282025 30291704

[4] Russell DA 2018 Sequencing, assembling, and finishing complete bacteriophage genomes. Methods Mol Biol 1681:109–125. doi:10.1007/978-1-4939-7343-9_9. 29134591

[5] Besemer J, Borodovsky M 2005 GeneMark: Web software for gene finding in prokaryotes, eukaryotes and viruses. Nucleic Acids Res 33:W451–W454. doi:10.1093/nar/gki487. 15980510 PMC1160247

[6] Delcher AL, Bratke KA, Powers EC, Salzberg SL 2007 Identifying bacterial genes and endosymbiont DNA with Glimmer. Bioinformatics 23:673–679. doi:10.1093/bioinformatics/btm009. 17237039 PMC2387122

[7] Cresawn SG, Bogel M, Day N, Jacobs-Sera D, Hendrix RW, Hatfull GF 2011 Phamerator: a bioinformatic tool for comparative bacteriophage genomics. BMC Bioinformatics 12:395. doi:10.1186/1471-2105-12-395. 21991981 PMC3233612

[8] Söding J, Biegert A, Lupas AN 2005 The HHpred interactive server for protein homology detection and structure prediction. Nucleic Acids Res 33:W244–W248. doi:10.1093/nar/gki408. 15980461 PMC1160169

[9] Rinehart CA, Gaffney BL, Smith JR, Wood JD 2016 PECAAN: Phage Evidence Collection and Annotation Network user guide. Western Kentucky University Bioinformatics and Information Science Center, Bowling Green, KY https://seaphages.org/media/docs/PECAAN_User_Guide_Dec7_2016.pdf.

[10] Altschul SF, Gish W, Miller W, Myers EW, Lipman DJ 1990 Basic local alignment search tool. J Mol Biol 215:403–410. doi:10.1016/S0022-2836(05)80360-2. 2231712

[11] Laslett D, Canback B 2004 ARAGORN, a program to detect tRNA genes and tmRNA genes in nucleotide sequences. Nucleic Acids Res 32:11–16. doi:10.1093/nar/gkh152. 14704338 PMC373265

[12] Russell DA, Hatfull GF 2017 PhagesDB: the actinobacteriophage database. Bioinformatics 33:784–786. doi:10.1093/bioinformatics/btw711. 28365761 PMC5860397

